# Development of an Oxide Layer on Al 6061 Using Plasma Arc Electrolytic Oxidation in Silicate-Based Electrolyte

**DOI:** 10.3390/ma15041616

**Published:** 2022-02-21

**Authors:** Priya Jadhav, Arunkumar Bongale, Satish Kumar, Danil Yurievich Pimenov, Khaled Giasin, Szymon Wojciechowski

**Affiliations:** 1 Symbiosis Institute of Technology (SIT), Symbiosis International (Deemed University), Lavale, Pune 412 115, India; priyasjadhav2018@gmail.com (P.J.); satishkumar.vc@gmail.com (S.K.); 2 Symbiosis Centre for Applied Artificial Intelligence, Symbiosis International (Deemed University), Lavale, Pune 412 115, India; 3 Department of Automated Mechanical Engineering, South Ural State University, Lenin Prosp. 76, 454080 Chelyabinsk, Russia; danil_u@rambler.ru; 4 School of Mechanical and Design Engineering, University of Portsmouth, Portsmouth PO1 3DJ, UK; khaled.giasin@port.ac.uk; 5 Faculty of Mechanical Engineering, Poznan University of Technology, 60-965 Poznan, Poland

**Keywords:** plasma electrolytic oxidation method, Al 6061, graphene, metal oxide formation

## Abstract

The plasma electrolytic method is one of the techniques which can be used to form an oxide layer on the substrate material surface. This technique employs ion exchange by developing an electrolytic arc between the cathode and the anode. The strong bond at high temperatures promotes the formation of an oxide layer on the metal surface. The electrolyte composition has a strong influence on the metal surface characteristics. Hence, the addition of certain nanoparticles in an adequate amount can improve the surface properties like wear and corrosion resistance. In this study, a plasma electrolytic technique based on using a direct current and voltage approach is investigated. The plasma electrolytic technique is utilized to develop an oxide layer on the Al 6061 alloy substrate surface using a DC voltage input on a silicate-based electrolyte. The substrate surface is then investigated for the thickness of the oxide layer formed and the amount of carbon element absorbed, using the SEM and XRD analysis. The experimentation and the study of the results confirmed the presence of a substantial oxide layer on the surface. The influence of the process on the output parameters-direct voltage and electrode distance is studied with the significant changes obtained in the weight percentage of elements like C, Al, Si, and O as supported by SEM and EDAX analysis. Most changes occurred when using a 197 V and in the current range of 0.3 A to 1 A. This can be useful further to improve the mechanical properties of the metal alloy using the plasma arc oxidation method.

## 1. Introduction

Plasma electrolytic oxidation (PEO) can produce a dominant crystalline oxide layer on the substrate alloy surface with specific electrolyte composition. The use of mild alkaline electrolytes makes the process more environmentally friendly than hard anodizing in a strongly acidic environment. Also, the implementation of high voltage during the PEO process with the local plasma results in the desired electrical discharges to produce thick coatings and achieve good microstructural control [1]. During the electrochemical reaction, sparks induced by a local discharge last from a few hundred to a few microseconds [2]. The oxide layers with a wide range of thicknesses can be generated quickly and efficiently on the surfaces of metal components of various shapes and sizes [3]. These coatings possess low porosity and excellent interfacial adhesion [4]. Since the transportation sector has adopted light-in-weight metal alloys to manufacture its components and requires better surface plating, plasma electrolytic oxidation (PEO) has gotten significant attention [5]. For this purpose, researchers have expanded the technical meaning of PEO by diversifying the development pathways and including additional precursors.

Almost a decade after its first successful use, graphene remains a material for various technological applications due to its unique characteristics [3]. Researchers recognize graphene as a super-material because of its high strength, high surface-to-mass ratio, and superconducting properties [6]. However, it is yet to be proved as a viable electronics material. Graphene is used as a scaffold in cell-tissue engineering, as an active electrode in supercapacitors to power implantable biomedical devices, and as detectors in biosensors [7]. High-temperature studies on graphene allow the researchers to understand the nanostructures’ stability, behavior, and interactions with the substrate [8]. Analyzing these interactions aids in understanding the fundamental processes that control graphene development at high temperatures and thus can be explored to modify its properties [9].

Different studies on graphene show that the absorption rate of the graphene starts at a temperature of 40 °C The Al 6061 exhibits improved tribological characteristics in a silicate-based aqueous solution with Graphene as an ingredient [10]. The Graphene Oxide (GO) particles are dispersed in the solution at a specified configuration. Results revealed that the porous structure of the coating had a higher alumina content, which strengthened the mechanical characteristics of the material [11]. The addition of the GO increased the surface microhardness and maintained a low friction coefficient with good corrosion resistance [10]. The polarization resistance was also enhanced. Using Hammer’s method, the magnesium ions were functionalized with increased corrosion resistance as the current density, and negative polarization loop decreased. Ionic type absorption at the surface coating enhanced barrier characteristics GO, resulting in higher R values than the uncoated samples [12]. PEO modified the process to obtain Al_2_O_3_ ceramic coatings on the AA2024 alloy surface by PEO, emphasizing the adherence of the coating to the substrate joining face. The results indicated that the coatings improved surface roughness, hardness, and thickness [13].

Spark plasma sintering was used to create bimodal grain size Al 7075 alloys with different ratios of coarse and fine grains. Coarse grains dissolve faster in acidic NaCl solution than fine grains because of their larger size, higher alloying element content, and higher second phase area [14]. A higher reduction rate of hydrogen ions led to an increased corrosion rate in the cathodic second phase for the coarse grain structure [15]. The mixture of both grain sizes enhanced the micro-scale electrochemical heterogeneity of the alloy. Hence, the improved mixing percentage of grains in the metal matrix accelerated corrosion in the acidic NaCl solution. The aluminate-based electrolyte is proven to be the best for corrosion resistance due to the formation of volcano-like granules on the surface [11].

The influence of Graphene concentration on the PEO coatings, produced on D16T aluminum alloy for the silicate-based electrolyte, were studied. The findings revealed that the morphologies of graphene-coated coatings differed significantly depending on the manner of graphene incorporation [16]. The coatings Al_2_O_3_ and Al were split into a porous layer and a thick inner layer. Coating thickness grew non-linearly as graphene content increased [17]. The corrosion resistance of graphene coating was greatly enhanced. Binary electrolyte additives, such as (Na (PO)_3_)_6_ and (H_3_BO)_3_ was utilized in this study to produce MAO coatings with enhanced thickness and microstructure on 6061 Aluminum alloys [18]. Compared to the basic silicate electrolyte, the results revealed that the total impact of the binary additions might have modified the MAO coatings’ discharge properties and microstructure morphologies [19]. It was possible to create a thicker and more durable MAO coating, which was mostly made of Al_2_O_3_ phases [20]. According to the literature, silicate electrolytes are advantageous to the oxidation process. They encourage the development of phases that provide significant adhesive strength between the substrate and the oxide layer [21]. The addition of nanoparticles determines the barrier characteristics quality of the oxide layer and contributes to the increased corrosion resistance and microhardness of the surface layers [19,22]. Graphene is well known for the different surface hardening processes for improving mechanical characteristics. The silicate-based electrolyte is widely used for the oxidation process due to its alkaline nature [23]. Under a strong electric field, electrophoresis affects the mechanical and tribological properties of a material [24]. Hence graphene-added silicate-based electrolytes are useful for improving the mechanical characteristics of the Al 6061 alloy by the micro-arc oxidation process [25]. According to the existing literature, the influence of direct current parameters from the plasma arc technique has not been well investigated. As a result, it is selected as the foundation for the experiments in the planned research work. The addition of the graphene in the electrolyte had less effect on the thickness of the oxide layer formed [8,26]. The oxidation process can be optimized more, keeping in view other process parameters.

The purpose of this article is to study the effects of the direct current voltage on the PEO process on Al 6061 alloy using a silicate-based electrolyte with Graphene. The effect of the electrolyte composition and processing parameters on the growth of the coating and microstructure properties are investigated. The following sections include significant detail on the experimentations carried out.

## 2. Materials and Methods

The spectroscopic analysis (BAIRD-DV6) following ASTM E 451-14 standard of the material Al 6061 is conducted for the elemental compositions as shown in Table 1. It is observed that the material is within the given specifications.

### 2.1. Mechanics of Oxidation Process

The reactions during the oxidation process play an important role in formulating the surface characteristics [27]. The Al metal ion exchange and reaction during the interaction has a significant value in terms of duration of process and composition of the material [28]. The dissolution and oxidation of metals are prime reactions in the plasma electrolytic oxidation process. The formation of the metal oxide is characterized by the following reactions at anode and cathode, respectively [29].

At anode:

(1) $$2\mathrm{Al}+3H_{2}O\to {\mathrm{Al}}_{2}O_{3}+6H^{+}\;$$

(2) $$2\mathrm{Al}\to {\mathrm{Al}}^{3+}+3e^{-}\;$$

The electrode gets oxidized when the decomposition of the electrolyte elements starts, and the metal oxide layer forms on the anode as per Equation (3).

(3) $$4{\mathrm{OH}}^{-}\to O_{2}↑+2H_{2}O+4e^{-}\;$$

### 2.2. Configuration of the Experiment

**Figure 1:**
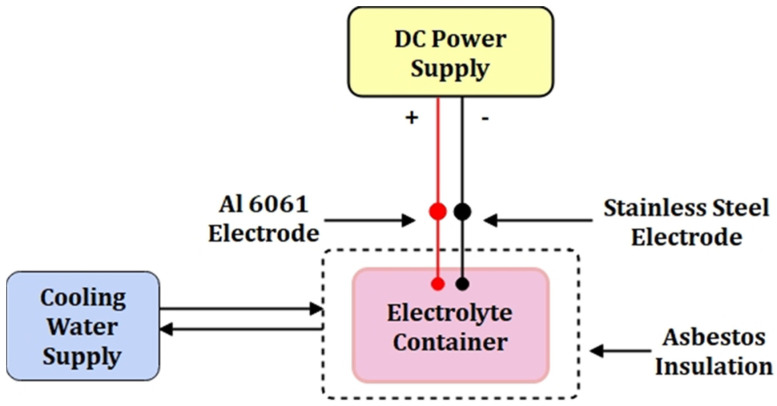
Schematic diagram of micro-arc oxidation set-up.

The experiments were conducted in the lab with configured set-up for micro-arc oxidation. The lab-based set-up consists of the electrolyte, electrode, container with asbestos insulation and cooling water circulating arangements, direct current (DC) power supply, stirrer, water inlet, and outlet with the constant water flow. The DC power source generates a high amount of heat and is used as the power input. The temperature inside the electrolytic container can be maintained low using the coolant or water flow around it, as shown in Figure 1.

The electrolytic chamber consists of a stainless-steel container with insulation and cooling water circulating through copper tubes. The electrodes supply the DC power up to 200 V. The cooling water maintained the electrolyte temperature at a constant low level. Al 6061 substrate represents the anode, while stainless steel represents the cathode. The compositions of the electrolytes NaOH and Na_2_SiO_3_ are shown in Table 2.

The geometry of the electrolyte chamber consists of an outer chamber and an inner chamber. The inner chamber comprises stainless steel with internal asbestos insulation 50 mm in thickness, and an outer chamber (550 mm × 400 mm × 400 mm) is a rectangular box. The insulation contains copper tubes for the fluid flow (coolant or water). The cooling arrangement is necessary to keep the temperature at lower values. Asbestos ensures the safety of handling the equipment during the operating condition. The stirrer is mounted inside the container during the process. The continuous movement of the electrolyte can reduce the agglomerate of masses.

During the experiments, 6061 Al alloy acts as a substrate, with an alkali silicate-based electrolyte containing graphene as an additive in the PEO process with one variable at a time approach. The coating condition was evaluated for the significant growth layer (i.e., optimized electrolyte concentration, current density, and process duration) on the surface of the substrate. In an earlier literature review for most of the cases, the electrolyte container was considered a cathode. The current PEO setup is different in the view of using the separate anode-cathode arrangement. This helps to understand the effect of electrode distance parameter and its significance in arc formation pattern due to lesser gap. The lower distance in the electrode is beneficial for strong arc formation resulting in a significant oxide layer.

During the experimentation, the composition of the electrolyte was kept constant. The primary purpose of the research was to identify the effect of the DC power source and additive absorption on the surface of the Al 6061 alloy during the oxidation process. The oxidation initiates with the plasma arc formation by connecting the two electrodes for ion exchange. A stronger arc promotes the metal ion exchange to form an oxide layer on the substrate [30]. Hence the varying parameter was taken distance between the electrodes. Experiments for each configuration were repeated three times, and the average values of the output parameters were obtained.

The experiments were conducted with electrolyte composition Na_2_SiO_3_: NaOH as 1:4.5 as shown in Table 2 and Table 3. In addition to this, a few experiments were also conducted using additive particles of graphene in 2 g/L concentration. The distance between the electrodes was maintained at 20, 30, 35, and 40 mm, respectively, to check its effect on the process output. The distance variation between the cathode and anode causes arc patterns and affects the graphene absorption on the surface layer.

The cuboidal samples of dimensions 100 mm × 10 mm × 10 mm were polished by gritted 800 # SiC sandpaper. The time of the coating process was taken as 20 to 40 min. The formation of the oxide layer and modified surface properties are discussed in the Result and Discussion section. The temperature measurement was carried out by a thermal imaging camera Testo 872, which can measure the temperature precisely at any point on the object. Hence, it is used to detect the temperature at different locations on the electrode in the electrolyte. It may help find out the heat transfer during the process. The sample surface microstructure was inspected with a Field emission scanning electron Microscope (FEI Nova NanoSEM 450, Make: JFEI company Of USA (S.E.A.), Hillsboro, OR, USA) The Energy Dispersive Spectrometer investigated the elemental distribution in the oxide layer formed on the substrate surface (EDS: Bruker XFlash 6I30, Make: Bruker Nano GmbH, Berlin, Germany). The thickness gauge was used to monitor the thickness of the oxide layer formed during the process. SEM examined a few samples for particle distribution, while FESEM examined the rest for cross-sectional development of the oxide layer on the substrate surface. Surface roughness was measured by Mitutoyo portable surface roughness tester (SURFESTEST- SJ-210 series, Make: Mitutoyo Europe GmbH, Neus, Germany) ISO 1997. The instrument gives Ra values for the surface. Three values for each face were taken before and after the coating process.

## 3. Results and Discussion

**Figure 2:**
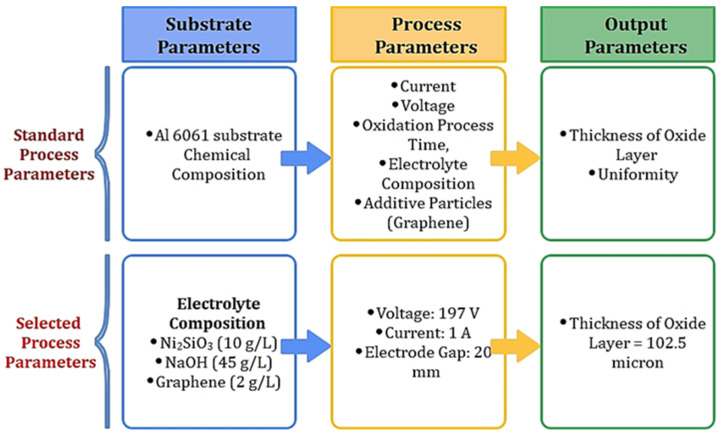
Output parameters after experimentation.

The experiments were conducted with the primary objective of coating the substrate surface with a uniform oxide layer using the Plasma Micro Arc Oxidation method for Al 6061 alloy. The efficiency of experiments conducted was evaluated with the help of various characterization tests, i.e., Scanning Electron Microscopic (SEM) Analysis and Field Emission Scanning Electron Microscopic (FE-SEM) analysis. The coating material, inclusion in the coatings, and oxidation properties were studied by Energy Dispersive Spectrometer (EDS) analysis, which gives elemental distribution on the sample surface [27,28]. The residual powder was also analyzed to identify the elements that did not adhere to the surface. The results are discussed in subsequent subsections. Figure 2 shows the results achieved and the values of the optimized parameters.

### 3.1. Formation of the Metal Oxide Layer

**Figure 3:**
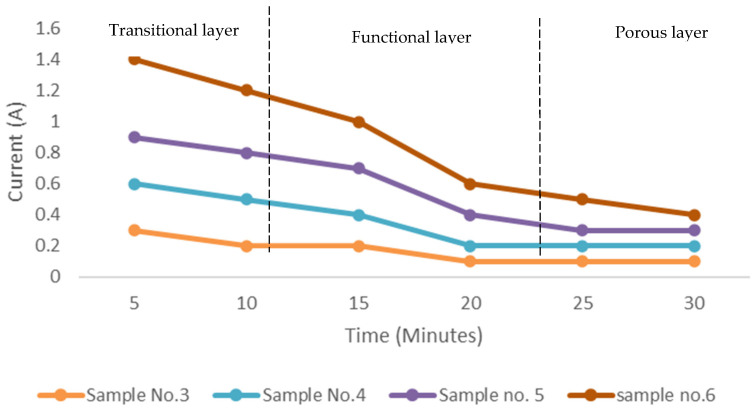
Current-time responses of samples processed at different electrode distances of 10, 30, 35, 40 mm.

It is observed that the metal oxide layer formed on the substrate surface depends on various input parameters. The experiments for the oxide layer formation using the micro-arc oxidation method were designed to study the effect of factors, i.e., operating current, potential difference between the electrodes (voltage), and the gap between the electrodes. The variation of these parameters and their effects on the oxide layer thickness is depicted in Figure 3. The oxide layer is formed without additive for some samples at 150 V and 197 V and different values of distance between electrodes. The graphene is also added to electrolytes for generating a few samples to study the effect on absorption.

It is observed that the current becomes stable after some time during the process. The reason is due to the formation of the metal oxide layer that acts as a barrier for the substrate material. It is observed that the growth of the oxide layer is due to the changes in the voltage and current values during the experimentation. The metal oxide layer forming on the substrate face at initial current values prevents the further exchange of the metal ions. Due to stabilization, the current starts reducing and becomes constant after some time and forms the outer layer. The sample with the lowest electrode gap, i.e., 20 mm, shows a drastic change in the current values.

**Figure 4:**
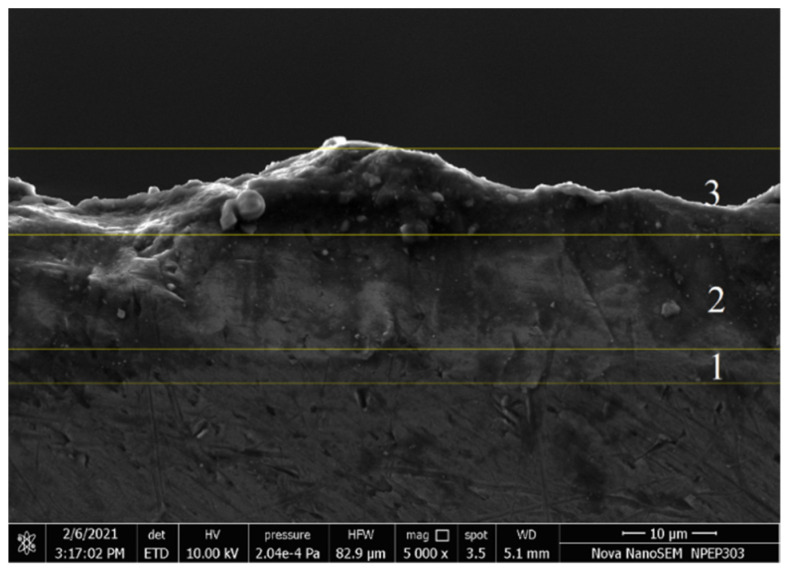
The formation of three characteristic layers in the PEO process. 1. Transition layer; 2. Functional layer; 3. Porous layer.

**Figure 5:**
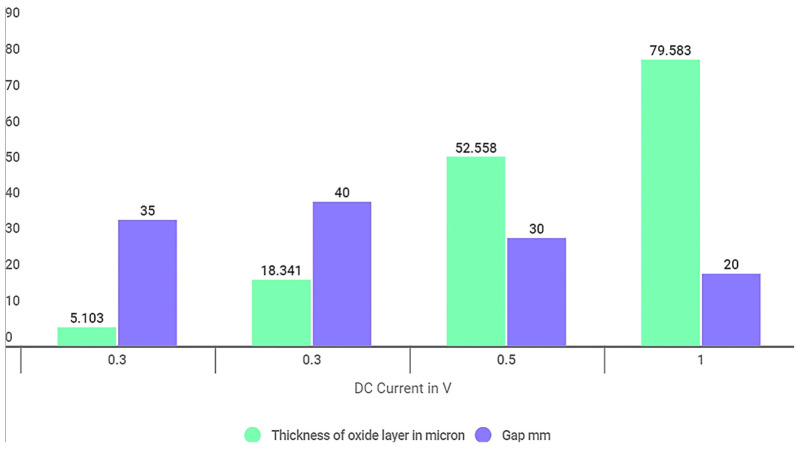
Effect of distance between electrodes and voltage on average oxide layer thickness during the PEO process.

Figure 4 indicates the formation of the oxidation layer on the substrate surface with three different regions in the PEO method. The transition layer, formed in the initial stage, provides a suitable platform for the functional layer. The range for each layer is approximately defined with respect to changes in current values, as a function of time. As the layer starts developing the current values are reduced and then stabilized after a certain time. The functional layer is the main layer of the coating formed during the PEO. The outermost layer is called a porous layer. It has a porous structure and is made of unevenly distributed oxides on the substrate. There is no formation of oxide layers on the substrate in the initial stages. So, the electrical conduction between electrodes is excellent, resulting in a significant potential difference between the electrodes. As the coating progresses with time, the oxide layer formed acts as an insulator, resulting in reduced potential difference and amperage between the electrodes. The increase in the oxide growth insulates the electrode and seizes the further development of the oxide layer. The oxide layer starts to develop after 20 min when the current stabilizes. This phenomenon indicates the relationship between the coating layer thickness with current and voltage. It is observed that the oxide layer thickness increased with an increase in the current [31]. With an increase in the current, the phase changes occur and mullite formation increases [13]. This was also supported by XRD images. At 0.3 A current, the oxide layer thickness was 5.1 μm. As the current value increased to 1 A, the oxide layer of thickness up to 79 μm was achieved. The effect of voltage on the oxide layer thickness is shown in Figure 3 and Figure 5.

**Figure 6:**
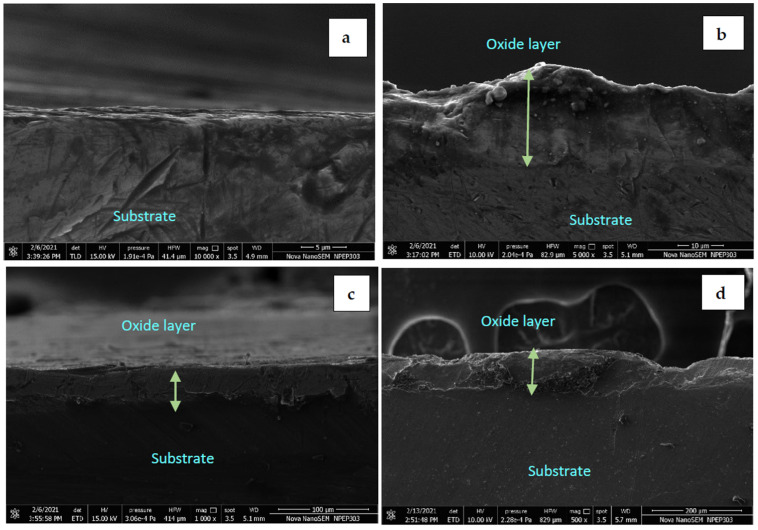
FESEM of the cross-section of the sample surface. (**a**) without forming an oxide layer. (**b**) At 197 V and 0.3 A. (**c**) At 197 V and 0.5 A. (**d**) At 197 V and 1 A.

Experiments were conducted with the electrode gap ranging from 20 mm to 40 mm. These experiments were repeated for different gap values. After the experiments, the thickness of the oxide layer formed was measured and documented as shown in Figure 6. It is observed that, at a gap of 20 mm between the electrodes, the process grew an oxide layer thickness of 80 μm approximately with a consumption of 1 A current. As the gap between the electrodes increased to 30 mm, the coating layer thickness reduced significantly as shown in Table 4.

Further increase in the gap distance resulted in a comparatively thinner oxide layer with reduced current amperage. Along the arc, the ion exchange occurs between the electrolyte and electrodes. This results in the growth of the oxide layer on the substrate. For the set of experiments conducted, the arcing between the electrodes was maximum at a 20 mm gap. As the gap increased, the arc efficiency and oxide layer thickness were reduced reducing the strength of the arc between the electrodes [32]. The energy consumed during the process depends upon the input parameters. The power increases with increases in the voltage resulting in greater reaction intensity. The input parameter increment enhances the formation of the uniform and dense coating [33].

### 3.2. SEM Analysis

The experiments were conducted to find the optimum ranges of the input parameters that can develop the significant layer of the oxide on the substrate during the PEO process. Hence, few samples were examined at the cross-section of the oxide layer to check the uniformity in the thickness. The experiments were conducted for more than 150 V as per the literature review reference [32,33]. Though literature used the AC voltage and current values, these values were taken as the base for DC values during experiments. It has been observed that the voltage values of 150–180 V were not sufficient to develop an oxide layer on the substrate surface. However, at 197 V, some layer thickness was visible, as shown in Figure 6. At current values 0.5 A and 1 A, the significant oxide layer is visible on a substrate surface. The uniformity and thickness of the oxide layer formed were measured at sample cross-sections using the FESEM tests. As 197 V was identified as the influencing voltage for the process, the microstructure at a configuration of 197 V and various current values was observed. At voltage values of 197 V and 0.3 A (Figure 6a) a minimal thickness was observed on the surface. As the electrode gap reduced, the strong arc formation promoted the growth of the oxide layer. Hence, the layer was thickened at a lower electrode gap and higher current values, as shown in Figure 6b,c. For current values up to 1 A, the oxide layer growth was up to 102.5 μm. The average oxide layer thickness developed is up to 79 μm as shown in Figure 6d. The layer became more prominent with the increase in voltage and current values [34,35].

**Figure 7:**
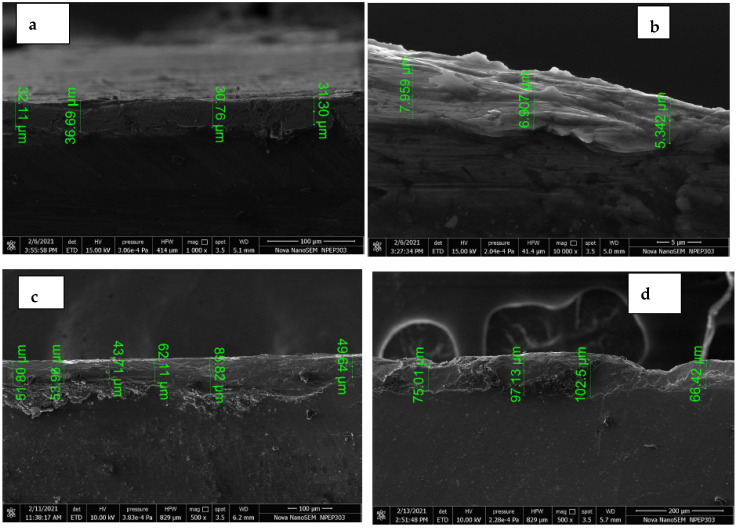
FSEM images for uniformity of the oxide layer at cross-section for an at (**a**) 197 V and 0.3 A. (**b**) At 197 V and 0.3 A. (**c**) At 197 V and 0.5 A. (**d**) At 197 V and 1 A.

The key area of this research was to study the effects of DC power input on the micro-arc oxidation process, as elaborated in the previous section. This oxide layer can be further analyzed to find the elements absorbed in the outer layer at the given input parameter configurations. It is observed that the percentage of graphene absorption increased at 197 V and current 0.3 to 1 A, as shown in Figure 7a. With current 0.3 A and 197 V, the oxide layer formed was very thin and not uniform, as shown in Figure 7b. With the increase in the current up to 0.5 A, the thickness improved up to 85.82 µm as shown in Figure 7c. The higher current values at 1 A formed a thicker and uniform layer in the range of 66.42 to 102.5 µm, as shown in Figure 7d.

**Figure 8:**
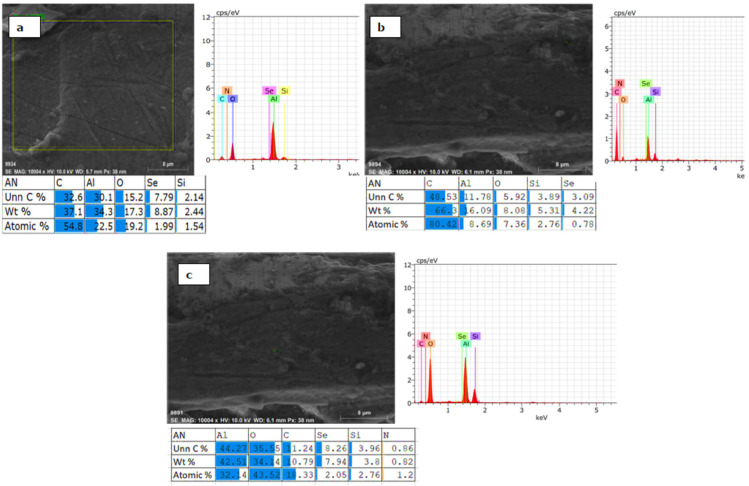
EDX images for samples (**a**) At 197 V and 0.3 A, (**b**) At 197 V and 0.5 A, (**c**) At 197 V and 1 A.

The experiment conducted at 197 V, 0.3 A and 197 V and 0.5 A showed enhancement in the percentage of the C element. The weight percentage of the C element was significantly improved from 10% to 66%, as shown in Figure 8a–c. It verifies the effect of the absorption of the graphene particles on the surface layer. Other elements like N, Si also varied significantly from 5% to 9% weight. The work done by Leonid et al. worked identified the PEO process for developing the 75 μm for the process duration of up to 180 min [36]. The use of Basalt salt in the Silicate based electrolyte in the PEO process produces 80 μm for 180 min duration [37]. The present research work is of the duration of up to 30 min with a significant average oxide layer developed up to 80 μm. The lower process duration as compared to the other work done until the date signifies the importance of the use of a DC power supply for the PEO process.

### 3.3. Effect of Temperature

**Figure 9:**
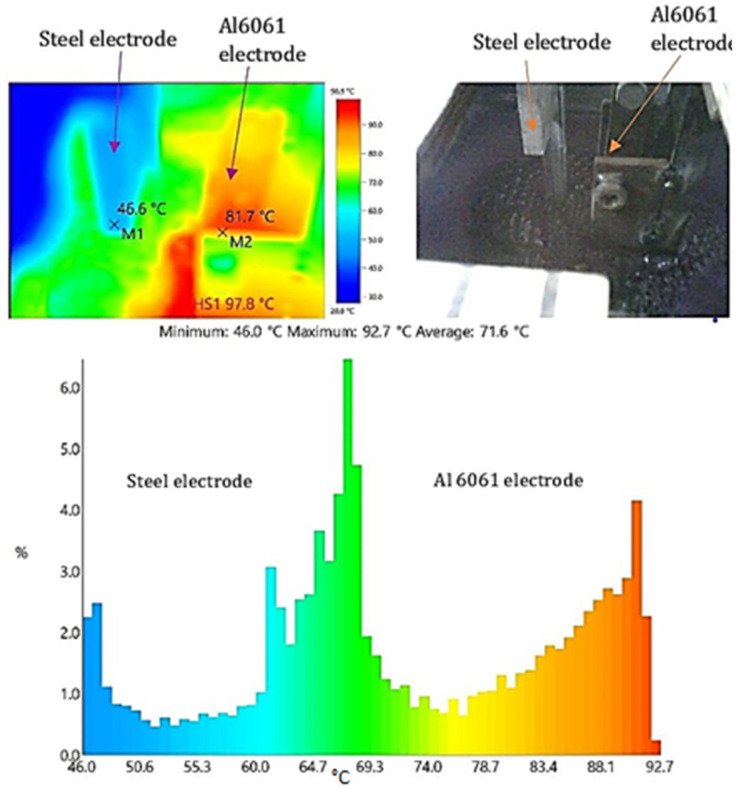
Temperature distribution between stainless steel electrode and Al 6061 electrodes.

During the experimentation, the temperature was measured using a thermal imaging camera. The temperature distribution in the electrolyte and across the electrode length was monitored. As per the temperature distribution shown in Figure 9, the variation in the temperature causes heat transfer between the two electrodes and the electrolyte. Maintaining a constant temperature was important for electrolytic composition and the uniformity of the oxide layer. Even though the temperature was increased gradually due to the heat generation, the cooling arrangement around the electrolytic chamber keeps the temperature variation at a minimum. This arrangement helped in achieving a more stable environment for the process. Hence, the electrolyte characteristics can be maintained constant throughout the coating layer formation.

### 3.4. Element Distribution on Al 6061 Surface

**Figure 10:**
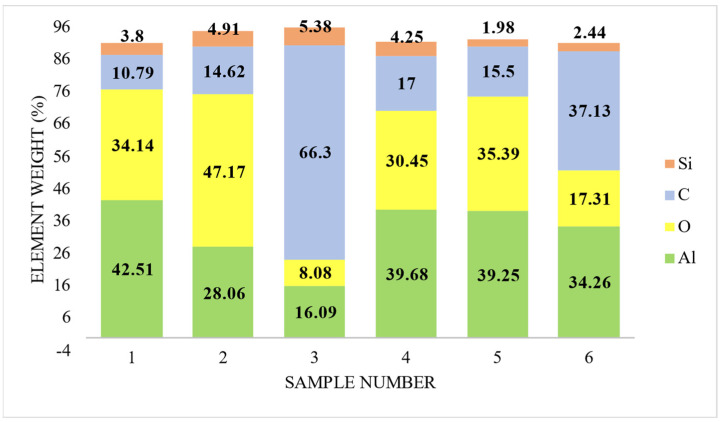
Variation in elemental percentage for samples.

The micrographs show the elements like Al, Si, C, O, and N as shown in Figure 8a. The varying percentage of oxygen indicates the oxide layer formed on the substrate. The uncoated Al 6061 has Al as 84.66%, which gets reduced to 11% when the input parameter was 197 and 0.5 A. On the other hand, the oxygen percentage was maintained between 25% to 3 %, as shown in Figure 8b. For most of the samples, traces of silicon and carbon can be found in Figure 8a. The additive particles of graphene get adhesive at higher temperatures. The percentage variation in the carbon element was between 28% and 66%. On average, the carbon particle absorbance was 34%, as shown in Figure 10. This indicates that the input parameter has a significant effect on the formation and growth of the oxide layer. The percentage variation in the atomic weight shows that the concentration of graphene in 10 g/L was incorporated in the outer oxide layer of the substrate. There may be some chemical compounds formed at the oxide layer that can be analyzed further in XRD images.

### 3.5. EDS Analysis of Residue

**Figure 11:**
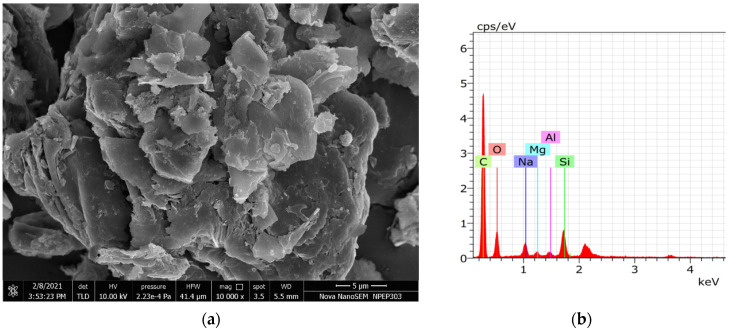
FESEM images and EDS analysis for the powder residue left after the Micro arc oxidation process. (**a**) FESEM image of the residue (**b**) element percentage of different elements in residue.

The SEM and EDS images show a change in element composition on the aluminum substrate surface due to the oxidation process, as shown in Figure 11. At the given input parameter, the oxide layer formation was in the initial stages for all the samples. Hence to study whether the chemical reactions were occurring at given current-voltage parameters and electrolyte composition, the residue after the process needs to be analyzed. The EDS of the residue showed different element particles like C, O, Na, Si which represents that the reactions were taking place at the higher amount of heat generated in the electrolyte during the oxidation process.

### 3.6. Phase Composition Analysis

**Figure 12:**
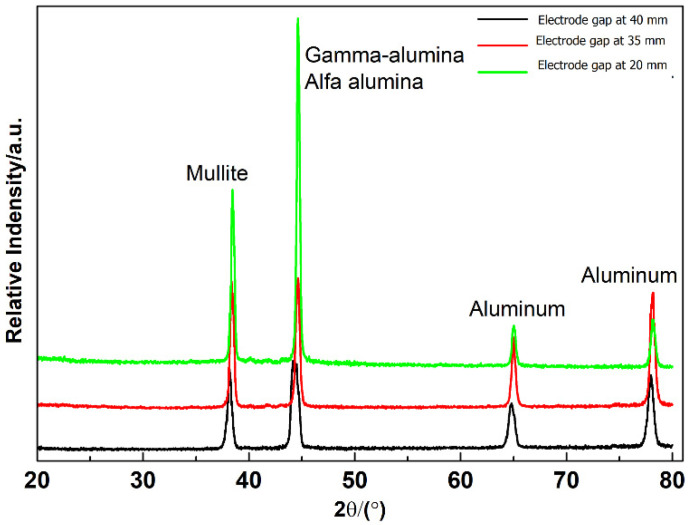
XRD for PEO coatings generated from the experimental configuration of 197 V and 20 mm, 35 mm, 40 mm electrode distances.

**Figure 13:**
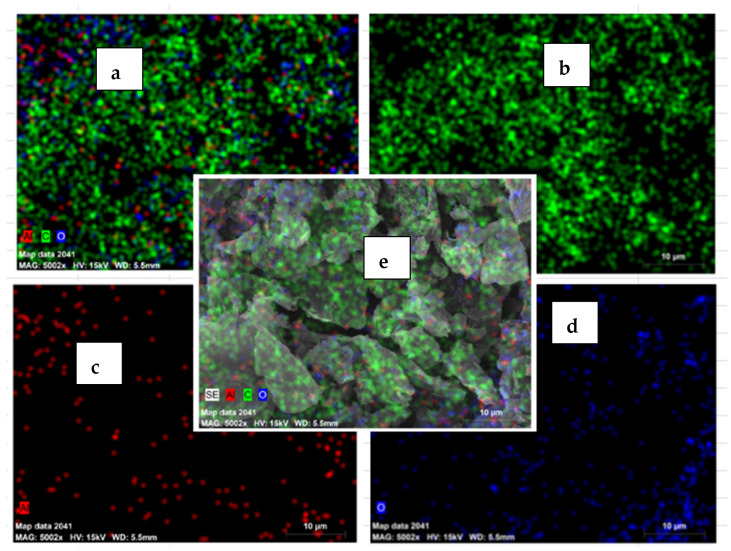
Elemental distribution SEM image for powder residue after experimentation. (**a**) Element distribution of Al, C,O, (**b**) Distribution of C (**c**) Distribution of Al, (**d**) Distribution of O, (**e**) Overall elemental distributions.

The phase analysis was carried out for samples with additives Graphene and produced from the PEO process in the silicate-based electrolyte. Figure 12 shows the XRD diffraction pattern for PEO coatings. As shown in Figure 12, the peaks of the mullite ($3{\mathrm{Al}}_{2}O_{3}\cdot 2{\mathrm{SiO}}_{2}$.) at the parameter configuration of 20, 35, 40 mm are visible. The other peaks are alpha-alumina $(\propto {\mathrm{Al}}_{2}O_{3}$) and gamma-alumina $\left({\gamma \;\mathrm{Al}}_{2}O_{3}\right)$ phases, as a visible insignificant amount. The highest amount of phase mullite is seen for the samples with an electrode gap of 20 mm. The alumina phases are visible as the prominent phase in XRD results. The sample processed with an electrode gap of 40 mm shows the lowest value, while the sample with a 20 mm electrode gap distance samples shows more significant phase changes of alumina. This occurs due to the strong arc formation between the electrodes which promotes the formation of different phases of alumina. The elementwise distribution is is as shown in the Figure 13.

### 3.7. Surface Roughness Analysis

**Figure 14:**
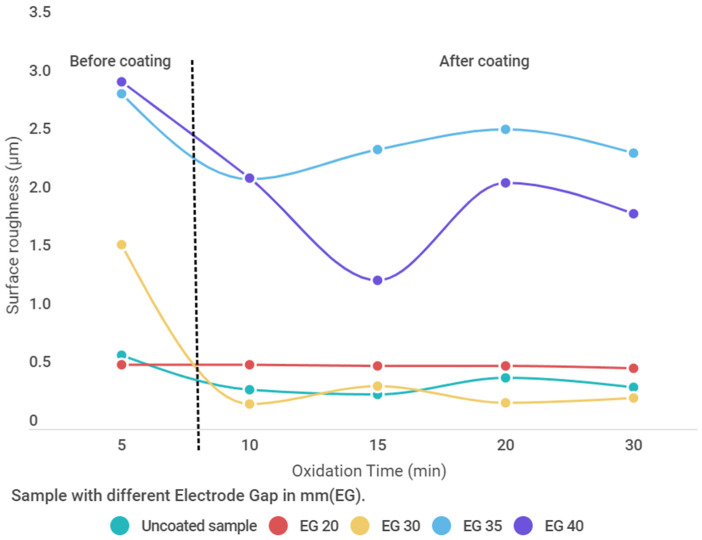
Surface finish values of the samples with Graphene addition in silicate-based electrolytes showing variation in surface finish values before coatings and after coating.

The most important parameter for the improvement of the substrate surface is roughness. It determines the contact resistance with the substrate material. A smooth surface with a uniform coating is characterized by a low coefficient of friction and low mechanical stress transfer [25]. The surface finish values are dependent on the voltage selection, electrolyte composition, and duration of the oxidation process. Figure 14 shows the surface roughness as a function of time. High discharge enhances crater formation resulting in a rough surface. The roughness values are lower for samples with an electrode distance of 20 mm and higher for 35 mm. The heat rise results in the local melting of elements in electrolytes present between the electrodes, and the solidification of the oxide layer occurs.

The silicate-based electrolyte when used by researcher Bosta et al. for the PEO process and AC voltage parameters showed the oxide layer thickness up to 18.5 μm with surface roughness up to 1.27 μm [38] with an average surface finish (0.3–0.4 μm). This supports the use of the DC power parameter for the PEO process for a good surface finish which requires less time.

## 4. Conclusions

This research work involves the use of the Plasma Electrolytic Oxidation technique to develop an oxide layer on the existing sample surface. The coating procedure is carried out on a sample made from Al 6061 alloy, using Graphene particles suspended in the electrolyte medium. The experiments were conducted using the direct current power supply as it is rarely reported in the literature. The variables in the experiments were voltage, current, and the gap between the electrodes.

The experiments were conducted with combinations of experimental variables and the samples were analyzed to evaluate the effect of the gap between electrodes, voltage & current, presence of Graphene Nanoparticles in the electrolyte, on the oxide layer formation. The results indicate substantial information about the process and variables.

It can be concluded that the oxide layer can be successfully developed on the Al 6061 samples using the silicate-based electrolyte with Graphene nanoparticle suspension during the PEO process. The Oxide layer consisted mostly of Alumina or Aluminum Oxide (Al_2_O_3_). The use of a direct power supply for the PEO process resulted in insufficient heat generation during the process and this has positive results due to the melting of solids in electrolytes between the electrode gaps. Hence more deposition occurs at higher temperatures and lower electrode gaps. From the study of SEM micrographs and EDS analysis, it is evident that there were very few traces of Graphene within the oxide layer. This concludes that irrespective of the Graphene content in the electrolyte, there is no significant Graphene absorption during the PEO process. There were significant changes in the percentage of elements like C, Al, Si, O as supported by SEM and EDAX analysis. Most changes occurred at 197 V DC and the current ranges from 0.3 to 1 A DC. The oxide layer formation was achieved up to a maximum of 200 μm in the cross-section of the samples with 197 V, 20 mm electrode distance, and 30 min oxidation time. The micro-arc oxidation process can be conducted at low temperatures through the controlled cooling of the electrolytic container. It helps to maintain the temperature at constant values avoiding excess heat generation. From the results of the conducted experiments, the optimized parameters obtained were 197 V and 1 A DC input, 30 min coating duration, and electrode gap of 20 mm generating a maximum of 102 μm of oxide layer on Al 6061 substrate in silicate-based electrolyte with Na_2_SiO_3_ (10 g/L), NaOH (45 g/L), and Graphene (2 g/L). The surface roughness values for samples show a rougher surface at the small distance between the electrodes. Hence at a 20 mm electrode distance, the surface roughness was higher than the electrode distance of 40 mm. It can be concluded that the gap of the electrode and surface roughness are inversely affecting each other.

## Figures and Tables

**Table 1:** Chemical composition of Al 6061 [22].

Element	Percentage (wt.%)	Specification Aluminum 6061
Cu	0.28	0.15–0.4
Mn	0.15	0.15
Mg	1	0.8–1.2
Zn	0.25	0.25
Cr	0.2	0.04–0.35
Ti	0.15	0.15
Si	0.6	0.4–0.8
Fe	0.7	0.7
Al	96.67	Remaining

**Table 2:** Input Parameter details of the PEO process.

Sr. No.	Parameter	Range
1	Voltage	150–200 V DC
2	Current	0–1 A DC
3	Time	5 to 15 min
4	Temperature	26–75 °C
5	Distance between two electrodes	35 to 50 mm
6	Additive	Graphene (2 g/L)
7	Electrolyte composition	Na_2_SiO_3_ (10 g/L), NaOH (45 g/L)

**Table 3:** Configuration of experiments.

Sample. No.	Electrolyte Composition	Voltage (V)	Time (min)	Electrode Gap (mm)
1	Na_2_SiO_3_ (10g/L), NaOH(45 g/L),	150	15	35
2		150	5	35
3	Na_2_SiO_3_ (10 g/L), NaOH (45 g/L), Graphene (2 g/L)	197	30	35
4		197	30	40
5		197	30	30
6		197	30	20

**Table 4:** The oxide layer thickness values as per variation in the distance between the electrodes.

Sr. No.	Distance between Electrodes in mm	The Thickness of Oxide Layer (µm)
1	20	79.58
2	30	52.56
3	35	5.10
4	40	18.34

## Data Availability

The data that support the findings of this study are available on request from the corresponding author (A.B.). However, the data is not available due to privacy/ethical restrictions.
